# Estimating the EQ-5D-5L value set for the Philippines

**DOI:** 10.1007/s11136-022-03143-w

**Published:** 2022-05-09

**Authors:** Red Thaddeus D. Miguel, Adovich S. Rivera, Kent Jason G. Cheng, Kim Rand, Fredrick Dermawan Purba, Nan Luo, Ma-Ann Zarsuelo, Anne Julienne Genuino-Marfori, Irene Florentino-Fariñas, Anna Melissa Guerrero, Hilton Y. Lam

**Affiliations:** 1 Institute of Health Policy and Development Studies, National Institutes of Health, University of the Philippines Manila, Manila, Philippines; 2 Institute for Public Health and Management, Feinberg School of Medicine, Northwestern University, Chicago, IL USA; 3 Social Science Department, Maxwell School of Citizenship and Public Affairs, Syracuse University, Syracuse, NY USA; 4 Health Services Research Centre, Akershus University Hospital, Lorenskog, Norway; 5 Department of Developmental Psychology, Faculty of Psychology, Universitas Padjadjaran, Jatinangor, Indonesia; 6 Saw Swee Hock School of Public Health, National University of Singapore, Singapore, Singapore; 7 Department of Health-Pharmaceutical Division, Manila, Philippines

**Keywords:** EQ-5D-5L, Hybrid mathematical modeling, Utility weights, QALY, Developing countries

## Abstract

**Background:**

The Philippines has recommended the use of Quality-Adjusted Life Years (QALYs) in government health technology assessments (HTA). We aimed to develop a value set for the EQ-5D-5L based on health preferences of the healthy general adult population in the Philippines.

**Methods:**

Healthy, literate adults were recruited from the Philippine general population with quota targets based on age, sex, administrative region, type of residence, education, income, and ethnolinguistic groups. Each participant’s preference was elicited by completing Composite Time Trade-Off (C-TTO) and Discrete Choice Experiment (DCE) tasks. Tasks were computer-assisted using the EuroQol Valuation Technology 2.0. To estimate the value set, we explored 20- and 8-parameter models that either use c-TTO-only data or both c-TTO and DCE (also called hybrid models). Final model choice was guided by principles of monotonicity, out-of-sample likelihood, model fit, and parsimony.

**Results:**

We recruited 1000 respondents with demographic characteristics that approximate the general population such as 49.6% Female, 82% Roman Catholic, 40% in urban areas, and 55% finished high school. None of the 20-parameter models demonstrated monotonicity (logical worsening of coefficients with increasing severity). From the 8-parameter models, the homoscedastic TTO-only model exhibited the best fit. From this model, mobility and pain/ discomfort had the highest effect on utilities.

**Conclusion:**

The selected model for representing the Philippine general population preferences for EQ-5D-5L health states was an 8-parameter homoscedastic TTO-only model. This value set is recommended for use in QALY calculations in support of HTA-informed coverage decisions in the Philippines.

**Supplementary Information:**

The online version contains supplementary material available at 10.1007/s11136-022-03143-w.

## Introduction

Health Technology Assessment (HTA) provides a transparent and rational priority setting mechanism for the optimal use of health technologies in a finite budget setting [1, 2]. Analyses in HTA often include economic evaluations which estimate the incremental cost-effectiveness ratios (ICERs) expressed as incremental cost per incremental benefits/outcomes/health effects. In recent years, the quality adjusted life years (QALY) has become a more common proxy measurement of health effects in HTAs and the EQ-5D-5L, a tool developed by the EuroQol Group [3] has become widely used as a means in quantifying changes in QALY due to an intervention. The EQ-5D-5L measures health-related quality of life using five dimensions (mobility, self-care, usual activities, pain/discomfort, and anxiety/depression) with each dimension having five levels (no problems, slight problems, moderate problems, severe problems, and unable/extreme problems) [4]. This tool has been translated into various languages and a number of national value sets have been published across the globe [5–17]. However, in order to utilize the EQ-5D-5L in QALY calculations, the five dimension scores of the tool need to be transformed into a single value through the use of a country-specific value set [3]. Despite using HTA for inclusion of drugs in the national formulary and recommending the use of QALYs in HTA, the Philippines has yet to establish its own value set [18].

In the absence of its country-specific value set, it has been a common practice for cost-effectiveness analyses (CEAs) in the Philippines to use either the Thai value set or the disability adjusted life years (DALY) [19–22]. The practice weakens the validity of these CEAs as there are socio-cultural differences between Thailand and the Philippines, which in turn could make the Thai value set an inappropriate proxy for the Philippine value set. In addition, DALY and QALY have theoretical differences that could lead to divergence in utilities [23], albeit a recent review has noted there might be minimal differences in HTA decisions due to these differences [24].

The enactment of the Universal Health Care Act in the Philippines (Republic Act 11223) in 2019 institutionalized the use of HTA to inform the coverage decisions of the Department of Health and the Philippine Health Insurance Corporation. Thus, establishing a Philippine-specific value set would be of high relevance to three of the five criteria in developing coverage recommendations applying HTA in the Philippines which are: (1) responsiveness to magnitude, severity, and equity of medical conditions with heaviest burden to the population; (2) cost-effectiveness; and (3) affordability and viability [25]. In this study, we aimed to estimate the utility values of EQ-5D-5L health states based on the preferences of the general population in the Philippines.

Over the years, several modeling approaches have been developed to generate country-specific value sets from empirical preference data [26, 27]. Earlier approaches used Composite Time Trade-Off (C-TTO) data only. However, this approach has been challenged as the iterative process could lead to biased responses due to responder fatigue, and both the hypothetical health states and time horizon could be difficult to visualize for the respondents [28]. Likewise, the use of conditional logit models to estimate coefficients using only Discrete Choice Experiment (DCE) data has had limitations observed in previous investigations [29]. Finally, recent EQ-5D valuation studies have found that in some contexts, the 20-parameter model produced coefficients that violate monotonicity (e.g., worse estimated decline if experiencing slight pain vs those experiencing severe pain). This is because additive models estimate a parameter for each domain level (e.g., a beta each for mobility levels 2 to 5). Recently, multiplicative models have been proposed which estimates fewer parameters and constructed in a way that avoids monotonicity violation [13, 30]. Thus, in this analysis, we likewise explored multiplicative models to generate the utility value set.

## Methodology

### Study design and sites

The Philippines is an archipelago with 17 administrative regions, wherein each region roughly follows the dominant local ethnolinguistic groups [31]. The study employed a cross-sectional design that was conducted in 34 towns across all the Regions in the Philippines (one rural and one urban town per Region). Data collection was conducted from October to December 2017.

### Sampling method and recruitment

Consistent with EuroQoL methodology, quota sampling based on 1000 respondents was employed in the study, with quota buckets calculated based on age, sex, administrative region, type of residence, education, income, and the six predominant ethnolinguistic groups (Tagalog, Cebuano, Ilocano, Hiligaynon, Waray, Bicolano) in order to produce a sample comparable to the general Philippine population [31]. Income was based on coverage under the National Household Targeting System (NHTS) which identified poor households based on a proxy means test [32]. This was selected because villages have lists of NHTS families which facilitated identification of potential respondents.

We included healthy, literate, and non-institutionalized adults (18 years or older) who provided consent. Healthy individuals were defined as respondents who did not self-report any disabilities or acute disease at the time of the survey. This was done through a screener that asked respondents ‘How do you feel today? Do you feel unwell? Do you have any illnesses?’ and ‘Do you have any disabilities?’.” Individuals who reported chronic diseases (e.g., hypertension, diabetes) were still included in the sample (26.6% reported chronic condition at time of survey). In each study site, the team coordinated with local government community health workers to identify individuals who met our inclusion criteria and these individuals were then invited to go to the recruitment area. Our study team members performed final screening before obtaining consent and conducting the final interview.

### Data collection

Three teams composed of three interviewers each were deployed. A supervisor was assigned to each team to ensure data quality.

Respondents who met the inclusion criteria were first asked to accomplish the informed consent. Thereafter, consenting respondents were interviewed by a trained interviewer fluent in their preferred language using a computer-based platform, EuroQol Valuation Technology (EQ-VT, version 2.0) software that followed the standard valuation protocol [33]. This version gave more attention to the valuation tasks than EQ-VT Version 1.1 and allowed respondents to review their responses through a new Feedback Module [17, 33]. Changes in version 2.0 including the revised quality control procedures and addition of the Feedback module has been found to improve data quality and consistency without affecting mean health state values [33–35].

Majority of the interviews were done in a room at the local government office, with a few being completed at health centers or at the respondent’s place of residence. Each respondent received a token worth PhP 150.00 (approximately USD 3.00) for the survey completion. Ethical clearance for this study, with protocol code UPMREB2017-156-01, was obtained from the University of the Philippines Manila Research Ethics Board.

### Instruments

#### EQ-5D-5L

The EQ-5D-5L is a multi-attribute health-related quality of life instrument with 3125 possible health states defined by its five dimensions (mobility (MO), self-care (SC), usual activities (UA), pain/discomfort (PD), anxiety/ depression (AD)) and five levels of severity (1 to 5, e.g., MO2 = slight problems with mobility). Thus, a five-digit number summarizes the level of problems for a specific individual. For example, health state ‘11111’ indicates no problem in any of the five dimensions [4]. The second part of the questionnaire is a vertical visual scale, called Visual Analog Scale (VAS), which records the respondent’s self-rated health on a scale of 0–100, where 0 means ‘the worst health you can imagine’ and 100 as ‘the best health you can imagine’.

The official Tagalog, Cebuano, and English language versions of the EQ-VT protocol were used. Translations were produced by the EuroQol Group using a standardized translation protocol that followed international recommendations [36].

#### EQ-VT interview

After obtaining informed consent, the team implemented the EQ-VT protocol consisting of five sections [11]:

1. General welcome and introduction to the study.
2. Completion of the self-reported EQ-5D-5L questionnaire and background questions (e.g., age, sex, experience of illness, disabilities, language proficiency, etc.).
3. Composite Time Trade-Off tasks commencing with a pre-test valuation of two wheelchair scenarios, followed by three scenarios of mild, moderate, and severe health states. It aimed to train respondents and to clarify their understanding. After which, valuation proceeded to 10 C-TTO tasks.
  The C-TTO uses traditional TTO to elicit better-than-dead (BTD) values and lead-time TTO to elicit worse-than-dead (WTD) values. This method is considered more robust than traditional TTO [28]. Details on C-TTO task can be found in Janssen et al. study [37]. There were 86 EQ-5D-5L health states included in EQ-VT for evaluation with C-TTO, distributed into ten blocks with similar levels of severity. Each block consisted of (i) one very mild state (only one dimension at level 2 and all others at level 1, e.g. ‘11112’), (ii) one most severe state (‘55555’), and (iii) eight intermediate health states. Respondents were randomly assigned to one of the ten C-TTO blocks, with each health state presented in random order [28].
4. Discrete Choice Experiment tasks wherein each respondent was randomly assigned to one of 28 DCE blocks with seven forced pair comparisons of health states. DCE has been included by the EuroQol Group to make valuation studies more robust and valid [38]. Respondents were presented with a pair of health states (i.e., *Life A is the health state at the left of the screen* and *Life B is the health state at the right of the screen*) to select their preferred state. The DCE design included 196 pairs of EQ-5D-5L health states distributed over 28 blocks, each consisting of seven pairs with similar severities. Further, the right-left order of the two health states were also randomized by the EQ-VT [14].
5. Feedback module where respondents were shown a rank order list of the c-TTO health states in the order of how severe they deemed the health state. The respondent would then have the option to flag specific health states if they felt that the order was incorrect. Flagged health states were excluded in the valuation computation. They were also asked about the difficulty of the c-TTO and DCE tasks using Likert scale questions, but this information was not used for the current analysis.

### Data quality control

The quality of data collected in an EQ-5D-5L valuation study relies heavily on interviewers’ skills and adequacy in explaining the C-TTO tasks [39]. The team hired interviewers with prior survey experience and had proficiency in Filipino, English, and at least one other major Philippine language (Cebuano, Bicolano, Ilocano, Hiligaynon, Waray). They underwent intensive training and received individual feedback before and during deployment.

During the actual fielding of the project, field supervisors provided on-site monitoring and feedback daily to their team. Additionally, the core team conducted bi-weekly meetings to address quality concerns. After the first 4 weeks of data collection, the team decided to conduct a 2-day retraining, as two interviewers were consistently flagged for 10–20% of the interviews they completed. After the retraining, none of the subsequent interviews were flagged.

### Statistical analysis

We explored various techniques previously used for modeling EQ-5D 5L valuation data which included TTO-only models, DCE-only models, and hybrid approaches which used both TTO and DCE. Hybrid approaches were known to address possible issues that may occur in models using C-TTO-only or DCE-only data. We still included non-hybrid approaches (e.g., TTO-only) to ensure comprehensive exploration of candidate models and consistency with prior practice in valuations in other countries [13, 14, 17]. More details in the modeling are provided in Ramos-Goñi et al. [27, 40]. The most widely used models contain either 20 or 8 parameters. The 20-parameter models (also called additive model) include a term for the effect of each level beyond the first level of each dimension (i.e., MO2 to MO5, SC2 to SC5, UA2 to UA5, PD2 to PD5, AD2 to AD5). This approach has been used in value sets, such as in Indonesia [14] and Germany [17]. The independent variables of the 8-parameter model (also called multiplicative model) include Level 5 utilities for each dimension (i.e., MO5, SC5, UA5, PD5, AD5) and the three intermediate utility levels (i.e., Level 2, Level 3, and Level 4). The same approach has been used in producing the Malaysian value set [8].

In selecting the final model to generate the value set, we first assessed the logical consistency of coefficients (i.e., the effect of severity levels increasing monotonically within each dimension). The next planned criteria applied were model fit and parsimony. While we tested many models, in this paper, we only present results from three 20-parameter approaches: (1) TTO-only 20-parameter Robust ordinary least squares (OLS); (2) TTO-only 20-parameter random intercept model; and (3) 20-parameter hybrid heteroscedastic model. We also explored various specifications of the 8-parameter models in terms of (1) data used (TTO-only vs hybrid), (2) intercept (fixed vs random), and (3) error (homoscedastic vs heteroscedastic). We compared the eight versions of the 8-parameter models using regular fit statistics and out-of-sample log-likelihood. All models were run using R 3.6.1. Hybrid and 8-parameter models were implemented using the ‘xreg’ package. Bootstrapping (10,000 samples) was used to estimate the confidence intervals for the 8-parameter model.

## Results

### Respondents’ characteristics

Among the 1107 individuals who were approached for the study, 1000 were included in the analysis. Among the excluded, 48 refused to participate, 30 did not meet inclusion criteria, and 29 were not included since the quota was already reached. (see Fig. 1 in Supplemental File 1). Respondents were given the choice for the interview site. Majority of the interviews were conducted in the local government unit offices, and several were at the respondents’ domicile or at the local health center. About a third of all respondents (34.7%), completed the study in a language other than English, Filipino, or Cebuano (the three languages available in the software).

The characteristics of the included respondents mirrored the Philippine general population in terms of age group, sex, ethnolinguistic group, and region. Unemployment rate was the only characteristic that showed greater than 10% absolute difference from the general population (13.7%). Although residence, education, and income, had some difference with the national estimates, the discrepancy with the targets were small (education: ± 2.3; residence: ± 5.3; income: ± 11.3) (Table 1).

**Table 1:** Demographic Characteristics of Respondents (*n* = 1000)

Demographics	*n* (%)	National %	Difference %
*Age group*			
18 to 30	330 (33.0)	33.0^a^	0.0
31 to 50	426 (42.6)	42.6^a^	0.0
51 and older	244 (24.4)	24.4^a^	0.0
*Sex*			
Male	504 (50.4)	50.4^a^	0.0
Female	496 (49.6)	49.6^a^	0.0
*Religion*			
Roman catholic	820 (82.0)	80.6^a^	+ 2.6
Aglipay	27 (2.7)	5.6^a^	− 2.9
Protestant	27 (2.7)	1.2^a^	+ 1.5
Iglesia ni Cristo	25 (2.5)	2.4^a^	+ 0.1
Islam	21 (2.1)	5.6^a^	− 3.5
Agnostic	3 (0.3)	0.1^a^	+ 0.2
Others	77 (7.7)	4.6^a^	+ 3.1
*Ethnolinguistic group*			
Tagalog	376 (37.6)	37.6^b^	0.0
Cebuano/Bisaya	277 (27.7)	27.7^b^	0.0
Ilocano	121 (12.1)	12.1^b^	0.0
Hiligaynon	101 (10.1)	10.1^b^	0.0
Bicolano	80 (8.0)	8.0^b^	0.0
Waray	45 (4.5)	4.5^b^	0.0
*Residential area*			
Urban	400 (40.0)	45.3^a^	− 5.3
Rural	600 (60.0)	54.7^a^	+ 5.3
*Region*			
I Ilocos region	50 (5.0)	5.0^c^	0.0
II Cagayan valley	24 (2.4)	3.4^c^	− 1.2
III Central Luzon	109 (10.9)	11.1^c^	− 0.2
IV-A CALABARZON	142 (14.2)	14.3^c^	− 0.1
IV-B MIMAROPA	27 (2.7)	2.9^c^	− 0.2
V Bicol region	58 (5.8)	5.7^c^	+ 0.1
VI Western Visayas	57 (5.7)	4.4^c^	+ 1.3
VII Central Visayas	90 (9.0)	6.0^c^	+ 3.0
VIII Eastern Visayas	44 (4.4)	4.4^c^	0.0
IX Zamboanga Peninsula	36 (3.6)	3.6^c^	0.0
X Northern Mindanao	49 (4.9)	4.6^c^	0.0
XI Davao Region	51 (5.1)	4.8^c^	0.0
XII SOCCSKSARGEN	45 (4.5)	4.5^c^	0.0
XIII CARAGA	26 (2.6)	2.6^c^	0.0
Autonomous region of muslim Mindanao (ARMM)	38 (3.8)	3.7^c^	+ 0.1
Cordillera administrative region (CAR)	26 (2.7)	1.7^c^	+ 1.0
National capital region (NCR)	128 (12.8)	12.8^c^	0.0
Education			
Finished high school	553 (55.3)	57.6^a^	− 2.3
Did not finish high school	447 (44.7)	42.4^a^	2.3
*Income*			
NHTS^f^	226 (22.6)	33.9^d^	− 11.3
Non-NHTS	774 (77.4)	66.1^d^	+ 11.3
Employment			
Employed	848 (84.8)	93.7^e^	− 8.9
Unemployed	152 (15.2)	6.3^e^	+ 8.9
Mean number of Individuals in Household	5.4 (2.39)	4.6^a^	+ 0.8
*Self-rated health category*			
Very good	162 (16.2)	N/A	N/A
Good	530 (53.0)	N/A	N/A
Fair	304 (30.4)	N/A	N/A
Bad	4 (0.4)	N/A	N/A
Average VAS Score	89.26 (SD: 8.4)	N/A	N/A

*VAS* visual analog score from EQ -5D-5L

^a^2010 Census of Population and Housing (CPH), Philippine Statistics Authority

^b^2000 Census of Population and Housing (CPH), Philippine Statistics Authority

^c^2015 Census of Population and Housing (CPH), Philippine Statistics Authority

^d^National Household Targeting System, Department of Social Welfare and Development (2016)

^e^2015 Annual Labor and Employment Status, Philippine Statistics Authority

^f^NHTS is the National Housing Targeting System which is a proxy measure for socio-economic status. Those under it are usually poor or have limited financial resources

Results further showed consistency between the reported health status and VAS score wherein those who reported ‘Very Good’ health state had the highest mean VAS Score (95, SD ± 6.8), while the lowest mean VAS score (82, SD ± 6.8) was noted among those reporting “Bad” health state (Table 1).

### Feedback module results

Each of the 1000 respondents valued 10 health states, providing 10,000 C-TTO observations. Of these, 1164 (11.64%) health state values were ‘flagged’ by the respondents themselves as being in incorrect order of health states severity during the Feedback Module task and were excluded.

In the DCE dataset, respondents completed seven paired comparisons of health states, providing 7000 DCE observations. Of these, 42 (4.2%) respondents were flagged for displaying unusual response patterns (e.g., AAAAAAA, BBBBBBB, ABABABA or BABABAB). These observations were included in the final analysis since our inquiry showed no indication of false responses.

### Modeling results

The three 20-parameter models showed non-monotonicity (Table 2) and were removed as candidates for final models to calculate the Philippine value set. TTO-only models showed inconsistency in the coefficients for the mobility dimension wherein Level 3 had lower coefficients than the Level 2. The TTO-only 20-Parameter Robust OLS model showed inconsistency for the Level 3 pain/discomfort dimension. Similarly, the 20-Parameter Hybrid Heteroscedastic Model yielded lower coefficients for Level 3 severity compared to Level 2 severity for all dimensions except for ‘usual activities’ (Table 2).

**Table 2:** Parameter of 20-Parameter Models to estimate Health State Utilities from EQ-5D-5L VT survey responses, Philippines, 2017

Parameters	TTO-only 20-parameter robust OLS		TTO-only random intercept model		Hybrid (DCE and TTO) heteroscedastic 20-parameter model^a^	
	Coefficient (*β*)	Std error	Coefficient (*β*)	Std error	Coefficient (*β*)	Std error
MO2	0.006	0.009	0.021	0.010	0.062	0.004
MO3	− 0.030^b^	0.009	0.004^b^	0.010	− 0.004^b^	0.007
MO4	0.113	0.010	0.161	0.011	0.153	0.009
MO5	0.273	0.009	0.300	0.010	0.102^b^	0.009
SC2	0.033	0.008	0.031	0.010	0.059	0.004
SC3	0.034	0.010	0.045	0.011	0.000^b^	0.006
SC4	0.165	0.010	0.196	0.011	0.139	0.008
SC5	0.294	0.009	0.292	0.010	0.052^b^	0.008
UA2	0.034	0.009	0.036	0.010	0.069	0.004
UA3	0.076	0.009	0.066	0.011	0.003	0.006
UA4	0.156	0.010	0.178	0.011	0.112	0.008
UA5	0.269	0.009	0.258	0.010	0.044	0.008
PD2	0.042	0.008	0.047	0.009	0.054	0.004
PD3	0.041^b^	0.010	0.063	0.011	0.001^b^	0.007
PD4	0.237	0.009	0.279	0.010	0.185	0.008
PD5	0.360	0.010	0.343	0.011	0.041^b^	0.009
AD2	0.028	0.009	0.016	0.011	0.060	0.004
AD3	0.041	0.010	0.059	0.012	0.026^b^	0.007
AD4	0.119	0.009	0.133	0.011	0.060	0.008
AD5	0.217	0.009	0.214	0.010	0.025^b^	0.007
CONS	0.028	0.009	0.029	0.012	0.012	0.004

^a^Other parameters are Intercept 2: 0.102 (0.006), sigma intercept: 0.047 (0.002), sigma slope 0.444 (0.007), and theta: 4.996 (0.152)

^b^Coefficients that break monotonicity (e.g., MO3 should be higher than MO2) pattern within the domain, *MO* mobility, *SC* self-care, *UA* usual activity, *PD* pain and discomfort, *AD* anxiety and depression, *CONS* constant. *TTO* time trade-off, *DCE* discrete choice experiment, *OLS* ordinary least squares

Among the 8-Parameter models, we chose the homoscedastic 8-parameter TTO-only model with random intercept as the final model. We observed that including a random intercept term improved out-of-sample log-likelihood without significantly changing the fit statistics like MSQE and ICC (see Table 2 to 5 in Supplemental File 1). The inclusion of DCE data through hybrid models or using heteroscedastic errors slightly improved log-likelihood but did not always improve fit statistics. Since the various random intercept models had similar fit statistics, we opted for the most parsimonious model to generate the Philippine EQ-5D-5L value set (Supplemental File 2). Given the non-normal nature distribution of the utility values, bootstrapping was used to generate 95% confidence intervals of the coefficients obtained (Table 3).

**Table 3:** Parameter of 8-parameter homoscedastic TTO-only model (preferred model)^c^ to estimate Health State Utilities from EQ-5D-5L VT survey responses, Philippines, 2017

Parameters	Coefficient (*β*)^a^	Std error	BS CI LL^b^	BS CI UL^b^
INTERCEPT	0.0211	0.0071	0.0070	0.0349
MO	0.3021	0.0090	0.2843	0.3201
SC	0.2879	0.0091	0.2699	0.3056
UA	0.2471	0.0095	0.2286	0.2652
PD	0.3677	0.0093	0.3493	0.3858
AD	0.2031	0.0084	0.1864	0.2198
L2	0.1331	0.0147	0.1043	0.1623
L3	0.1668	0.0137	0.1396	0.1937
L4	0.6966	0.0144	0.6682	0.7246
log(σ)	− 1.3347	0.0128	− 1.3603	− 1.3106
log(ω)	− 1.7338	0.0415	− 1.8203	− 1.6592

Example estimated values by health state	Unadjusted	Adjusted
Utility 11111	0.9789	1.000
Utility 12345	0.4402	0.4423
Utility 55555	− 0.4289	− 0.4381

^a^Estimated coefficients are statistically significant with *p* values less than 0.05

^b^Bootstrapped mean, upper and lower limit confidence intervals based on 2.5% and 97.5% percentiles of 10,000 samples

^c^The equation for the preferred model is as follows:$$\begin{aligned} & y = \alpha + \left( {\beta _{{{\text{MO}}}} x_{{{\text{MO2}}}} + \beta _{{{\text{SC}}}} x_{{{\text{SC2}}}} + \beta _{{{\text{UA}}}} x_{{{\text{UA2}}}} + \beta _{{{\text{PD}}}} x_{{{\text{PD2}}}} + \beta _{{{\text{AD}}}} x_{{{\text{AD2}}}} } \right)L_{2} + \\ & \left( {\beta _{{{\text{MO}}}} x_{{{\text{MO3}}}} + \beta _{{{\text{SC}}}} x_{{{\text{SC3}}}} + \beta _{{{\text{UA}}}} x_{{{\text{UA3}}}} + \beta _{{{\text{PD}}}} x_{{{\text{PD3}}}} + \beta _{{{\text{AD}}}} x_{{{\text{AD3}}}} } \right){\text{L}}_{3} + \\ & \left( {\beta _{{{\text{MO}}}} x_{{{\text{MO4}}}} + \beta _{{{\text{SC}}}} x_{{{\text{SC4}}}} + \beta _{{{\text{UA}}}} x_{{{\text{UA4}}}} + \beta _{{{\text{PD}}}} x_{{{\text{PD4}}}} + \beta _{{{\text{AD}}}} x_{{{\text{AD4}}}} } \right){\text{L}}_{4} + \\ & \beta _{{{\text{MO}}}} x_{{{\text{MO5}}}} + \beta _{{{\text{SC}}}} x_{{{\text{SC5}}}} + \beta _{{{\text{UA}}}} x_{{{\text{UA5}}}} + \beta _{{{\text{PD}}}} x_{{{\text{PD5}}}} + \beta _{{{\text{AD}}}} x_{{{\text{AD5}}}} + {\text{e}} \\ \end{aligned}$$

Where e is an error term assumed to have a mean of zero and $x$ variables (e.g., *x*_MO2_^)^ are binary indicator variables of the responses so that an MO score of 4 means *x*_MO4_ = 1 and all other *x*_MO_’s are coded as 0. *MO* mobility, *SC* self-care, *UA* usual activity, *PD* pain and discomfort, *AD* anxiety and depression, *L* level, log(*σ*) is the estimated variance term for the error distribution; log(*ω*) is the error term of the respondent-level random intercept.

Based on the 8-Parameter Homoscedastic TTO-only Model, the health state utilities were computed using the formula: 1−(*β*_MO_ × *β*_Li_)−(*β*_SC_ × *β*_Li_)−(*β*_UA_ × *β*_Li_)−(*β*_PD_ × *β*_Li_)−(*β*_AD_ × *β*_Li_)−*α*, where *β*_MO_ represents dimension coefficient, β_Li_ level coefficient and α the intercept. Hence, health state ‘11111’ (full health) would be 1−0.0211 = 0.9789, which is the (unadjusted) maximum value. On the other hand, health state ‘44444’ will have unadjusted utility of 1−0.0211−[(0.3021 + 0.2879 + 0.2471 + 0.3677 + 0.2031) × 0.6966] = − 0.0017. Since the preferred model has a nonzero intercept that leads to a predicted value of less than 1.000 (i.e., 0.979 for the full health (‘11111’), the team decided to apply linear adjustment to all the health states. This was done by dividing the coefficients by 1−*α* [13] and using the adjusted coefficients (except the intercept) to calculate the utilities (see Supplemental Table 1 in Supplemental File 1). Therefore, the adjusted value for health state ‘11111’ becomes 1 representing full health and ‘44444’ becomes − 0.0234. Consequently, the most severe health state (‘55555’) value equated to − 0.4289 (unadjusted) and − 0.4381 (adjusted). (See Supplemental File 2 for calculated values for all health states).

## Discussion

In this study, we demonstrated the complexities of developing a value set in a multi-lingual country context while also creating an important resource to facilitate health technology assessment in the Philippines. We extended the literature for EQ-5D-5L valuation in several ways. First is that we showed an adaption of the protocol that allowed inclusion of speakers of languages that have not been included in the valuation software. Second, we presented the value of running multiple models covering additive and multiplicative approaches as well as using c-TTO-only and hybrid datasets. After running several models, we selected an 8-parameter TTO-only model with homoscedastic error term and a random intercept at the level of individual study respondents as the most appropriate model to generate the Philippine value set. We found that 20-parameter models violated the logical dominance order of the EQ-5D-5L descriptive system. Meanwhile, we found that c-TTO + DCE hybrid models did not significantly improve model performance. Third, we quantified the underestimation of utilities with use of value sets from a neighboring country rather than a country-specific value set. We found that the generated utility weights were, on the average, higher than those in the Thai value set suggesting, differences in health preferences between the Thai and Philippine populations.

**Fig. 1:**
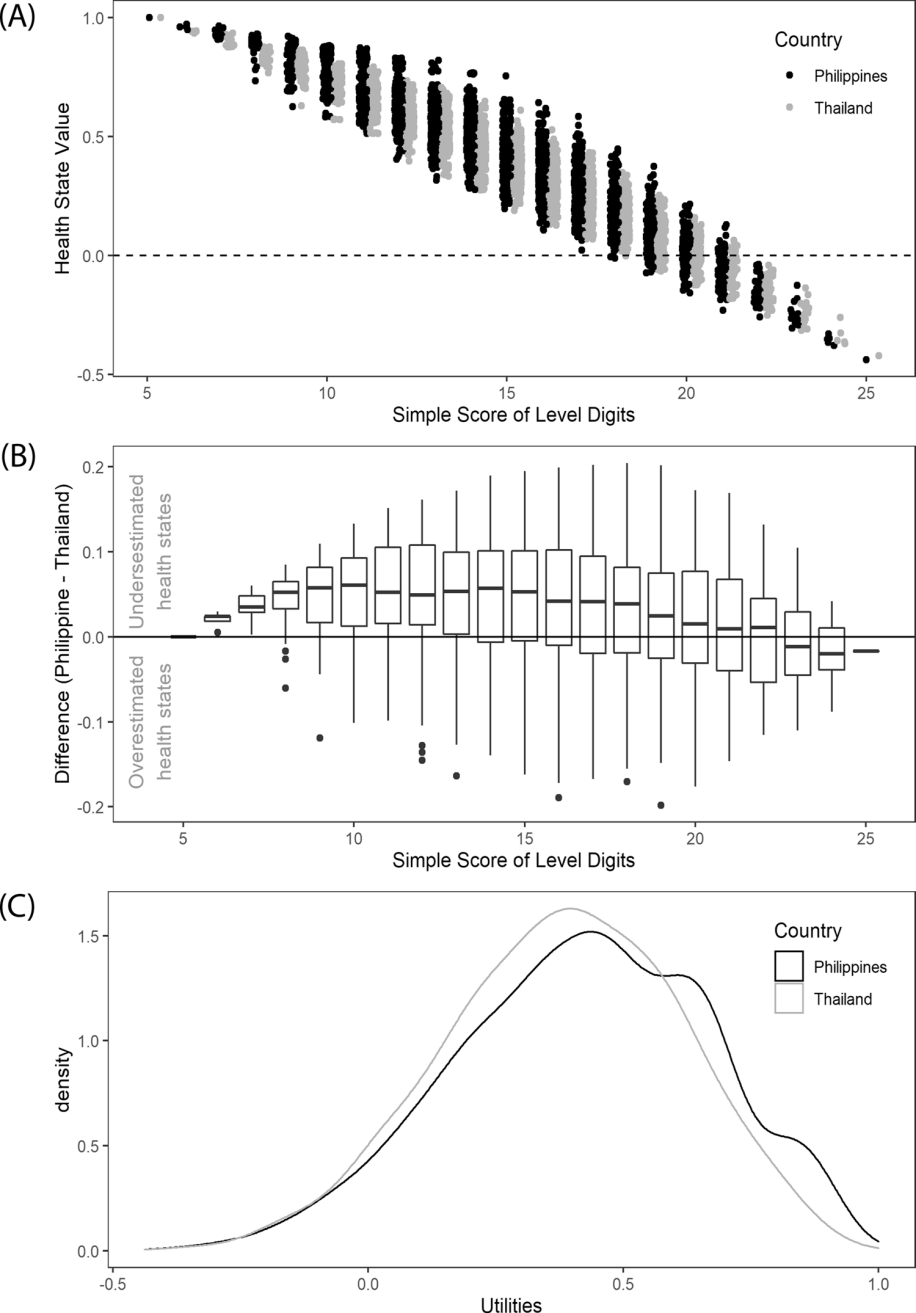
Comparison of the Philippine and Thai EQ-5D-5L value set: **A** density curve of utilities, **B** differences per simple score of level digits, **C** utilities per simple score of level digits

According to the final model, mobility and pain/discomfort are the two dimensions that have the highest impact on the utility, and 169 (5.41%) health states have negative values or are considered worse than death. While there is some overlap in the Philippine and Thai values set [7], the Philippine utilities skewed more toward one (1) than the Thai utilities (Fig. 1A). Most (72%) of the Philippine utility values were higher with an average difference of 0.041 points (SD: 0.072). This underestimation of Philippine values by the Thai value set is most severe in states with lower sum scores (Fig. 1B). For example, the utility for ‘12345’ (sum score of 15) for the Philippines was 0.4423 and for Thai it was 0.3685. At higher sum scores (and presumably worse states), the difference narrowed, and the Thai utilities then tend to overestimate the Philippine utilities at sum score of 23 and higher (e.g., at ‘55555’, Philippine utility is − 0.4381 while Thai is higher at − 0.4211). We also note that the Philippine value set seemed to have more variability within groups based on sums of level digits compared to the Thai value set (Fig. 1C).

Previous EQ-5D-5L valuation studies in the region used other regression models for their valuation. South Korea [12] used a variation of the TTO-only model while Japan [11], Hong Kong [16], Indonesia [14], and Thailand [7] used the 20-Parameter Hybrid Model. These models resulted in non-monotonic utility values when applied to the Philippine data. One example is that individuals having moderate problems with mobility would have higher health utility than those having slight problems with mobility. Simplified non-linear models have also been proposed as these are more parsimonious and, in the other value sets, have been demonstrated to outperform the 20-parameter model in terms of predicting out-of-sample health states [30]. The 8-Parameter model was one of the new approaches and was first used in the Malaysia EQ-5D-5L valuation study [8]. In our case, we found that using the hybrid approach did not lead to much better fit to the data compared to using only TTO data.

Additionally, our results demonstrated that Filipinos value each domain differently and have different overall health preferences compared to other populations. Our results suggested that the ‘mobility’ dimension had the highest impact on health-related quality of life, followed by the ‘pain/discomfort’, ‘self-care’, ‘usual activities’, and ‘anxiety/depression’ dimensions. This is consistent with the reporting from 75% of the respondents that self-care and mobility are more important considerations in completing the DCE tasks (see Supplemental Table 6 in Supplement File 1). Mobility also had the highest utility estimates in South Korea [12], Japan [11], Canada [6], Uruguay [10], Indonesia [14], and Thailand [7]. On the other hand, ‘pain/discomfort’ and ‘anxiety/depression’ have higher utility estimates in Netherlands and England [5, 9] and this might be related to more accessible living conditions and less emphasis on manual labor in these countries. Future studies, especially qualitative ones, are needed to explore reasons for these observed differences, especially those between the Philippines versus surrounding nations like Thailand and Indonesia.

While our study is the first nationwide valuation study for the Philippines, it has several limitations. The main limitation was the use of a non-probability-based sampling design, which may have affected external validity and made it less likely to produce a statistically representative sample. To minimize this, the team obtained a sample that was roughly like the national general population in key demographic characteristics using a quota system. Another limitation was that we excluded illiterate individuals, albeit by necessity. While this group comprises only a minority of Filipinos (4.4%) [41], we are unable to assume that they hold the same preferences as the literate population. Another limitation was that the valuation software was translated only to English, Filipino, and Cebuano despite having at least four other major languages in the sites visited. The translation ambiguity or inaccuracy may have been the reason for non-monotonic coefficients present in regression models other than the chosen 8-parameter hybrid model [14]. We mitigated the impact of translation ambiguity in multiple ways. First, we asked participants to select which of the three available languages they feel most comfortable in using. Second, we recruited interviewers who are fluent in the non-translated major languages and provided interviewers standardized translations of the EQ-5D-5L instrument which allows them to describe the various health states in the languages not available in the software. Finally, to ensure minimal biases and variability during data collection, we coordinated closely with the EuroQol foundation in adapting the EQ-5D-5L data collection and valuation protocol for the Philippine context and implemented the quality control process recommended by the foundation for valuation studies. While we followed the current EQ-5D valuation protocol [33], the use of the feedback module resulted in flagging and dropping of data. Our rate (11%) is also at the higher rate among published flagging rates (4.3% in Norway [35] to 9.7% in Indonesia [14]). We view the exclusion of these data points as an important trade-off to improve consistency and facilitate modeling of the data. We are also unable to examine the influence of socio-demographic characteristics on odds of flagging. These questions are important for future development of EQ-5D-5L valuation protocol. Lastly, our sample only covered the adult population. While it may be acceptable for now to use this value set for HTA of interventions for children, future work on using and valuation of the EQ-5D-Youth is needed.

## Conclusion and recommendations

An 8-parameter TTO-only model with a homoscedastic error term was selected as the best representation of the Philippine general population preferences for EQ-5D-5L health states. This Philippine EQ-5D-5L value set is recommended for use in EQ-5D, and should be helpful in performing QALY-based economic evaluations to facilitate HTA-informed coverage decisions in the country. Future research is called for to explore the issues raised around translation ambiguity, the potential impact of these on utility valuation, and how to better account for such in subsequent modeling.

## Supplementary Information

Below is the link to the electronic supplementary material.

Supplementary file1 (DOCX 55 kb)

Supplementary file2 (XLSX 207 kb)

## Data Availability

The data are available upon request from the Department of Health-Pharmaceutical Division.
