# Supplementary material for: Estimating the EQ-5D-5L value set for the Philippines
Source: Qual Life Res. 2022 May 9;31(9):2763–74. doi: 10.1007/s11136-022-03143-w (PMC9356948; doi:10.1007/s11136-022-03143-w)
Supplement: Supplementary file 1 — Supplementary file1 (DOCX 55 kb) [file 11136_2022_3143_MOESM1_ESM.docx]

**Supplemental File 1 for**

**Estimating the EQ-5D-5L Value Set for the Philippines**

*Authors*: Miguel, Red Thaddeus D.^1^, Rivera, Adovich S.^2^, Cheng, Kent Jason G.^3^, Rand, Kim^4^, Purba, Fredrick Dermawan^5^, Luo, Nan^6^, Zarsuelo, Ma-Ann^1^, Genuino-Marfori, Anne Julienne^7^, Florentino-Fariñas, Irene^7^, Guerrero, Anna Melissa^7^, Lam, Hilton Y.^1^

*Affiliations:*

^1^Institute of Health Policy and Development Studies, National Institutes of Health, University of the Philippines Manila, Manila, Philippines

^2^Institute for Public Health and Management, Feinberg School of Medicine, Northwestern University, Chicago, IL, USA

^3^Social Science Department, Maxwell School of Citizenship and Public Affairs, Syracuse University, Syracuse, NY, USA

^4^Health Services Research Centre, Akershus University Hospital, Lorenskog, Norway

^5^Department of Developmental Psychology, Faculty of Psychology, Universitas Padjadjaran, Jatinangor, Indonesia

^6^Saw Swee Hock School of Public Health, National University of Singapore, Singapore, Singapore

^7^Department of Health-Pharmaceutical Division, Manila, Philippines

1. **Supplementary Methods**

*Quality Control*

A EuroQol prescribed “Quality Control” (QC) tool was utilized for the study. The QC tool is a Microsoft Excel-based program that automates the production of reports based on EQ-VT studies [37]. The QC tool reports provide quantitative indicators of interviewers’ compliance to the EQ-VT protocol. Specifically, the QC tool assesses how interviewers explain the questions as well as their overall conduct in using the composite time trade-off (C-TTO) tasks. An interview was ‘flagged’ as suspicious if any of the following indicators were observed: (i) the interviewer explained the ‘wheelchair examples’ in less than three minutes, (ii) the interviewer did not explain the lead-time C-TTO (WTD) in the wheelchair examples, (iii) the respondent completed the ten actual C-TTO tasks in less than five minutes, and (iv) the value for state ‘55555’ was not the lowest among the ten C-TTO health states being valued and it was at least 0.5 (5 years in C-TTO time-frame) point higher than that of the state with the lowest value. Additionally, suspicious pattern in responses to DCE tasks (i.e., the respondent always chose Life A, always chose Life B, or choices A and B seem to be alternating like ABABABA or BABABAB) would also be flagged.

*Recruitment Method*

**Supplemental Figure 1.** Recruitment and Final Data Set.

*Post-valuation Survey*

As part of the EQ-5D-5L valuation survey, participants were asked about their decision making during the time trade-off and discrete choice experiments. One of their questions relate to which domain(s) they considered when comparing the two health states with different lengths of life shown to them during the DCE task. The distribution of responses to this question are shown in Supplemental Table 6.

*8-parameter model and calculation of health states*

The 8-parameter model is described mathematically as follows:

$$y= \alpha+ \boldsymbol{\Sigma}_{l}\left( \boldsymbol{\Sigma}_{d}\boldsymbol{\beta}_{d}\mathbf{x}_{dl} \right)\mathbf{L}_{l}+e$$

$$y= \alpha+$$

$$\left( \beta_{MO}x_{MO2}+\beta_{SC}x_{SC2}+ \beta_{UA}x_{UA2}+ \beta_{PD}x_{PD2}+ \beta_{AD}x_{AD2} \right)L_{2}+$$

$$\left( \beta_{MO}x_{MO3}+\beta_{SC}x_{SC3}+ \beta_{UA}x_{UA3}+ \beta_{PD}x_{PD3}+ \beta_{AD}x_{AD3} \right)L_{3}+$$

$$\left( \beta_{MO}x_{MO4}+\beta_{SC}x_{SC4}+ \beta_{UA}x_{UA4}+ \beta_{PD}x_{PD4}+ \beta_{AD}x_{AD4} \right)L_{4}+$$

$$\beta_{MO}x_{MO5}+\beta_{SC}x_{SC5}+ \beta_{UA}x_{UA5}+ \beta_{PD}x_{PD5}+ \beta_{AD}x_{AD5}+e$$

Using the coefficients from the results, health state 22222 will be computed as 1 – {(0.3021 + 0.2879 + 0.2471 + 0.3677 + 0.2031) x 0.1331] – 0.0211} = 0.7915. Given that health state 11111 refers to 1 – 0.0211 = 0.9789, values are in turn normalized allowing 11111 to equate to 1 and consequently 22222 transforms in value to 0.8014. Below are the coefficients for calculating the rescaled values.

**Supplemental Table 1.** Re-scaled coefficients to calculate Re-scaled Utilities

| Parameters | Coefficient (β)^a^ | Std Error | BS CI LL^*^ | BS CI UL^*^ |
| --- | --- | --- | --- | --- |
| MO | 0.3086 | 0.0092 | 0.2906 | 0.3271 |
| SC | 0.2941 | 0.0091 | 0.2761 | 0.3118 |
| UA | 0.2524 | 0.0091 | 0.2346 | 0.2700 |
| PD | 0.3756 | 0.0098 | 0.3563 | 0.3948 |
| AD | 0.2075 | 0.0086 | 0.1906 | 0.2245 |
| L2 | 0.1360 | 0.0143 | 0.1077 | 0.1640 |
| L3 | 0.1704 | 0.0137 | 0.1432 | 0.1972 |
| L4 | 0.7116 | 0.0154 | 0.6812 | 0.7416 |
| LN_SIGMA | -1.3634 | 0.0172 | -1.3980 | -1.3301 |
| LN_OMEGA | -1.7711 | 0.0437 | -1.8631 | -1.6921 |

*Notes: ^*^Bootstrapped mean, upper and lower limit confidence intervals based on 2.5% and 97.5% percentiles of 10,000 samples*

*^a^ Estimated coefficients are statistically significant with p-values less than 0.05. MO – mobility, SC – self-care, UA – usual activity, PD – pain and discomfort, AD – anxiety and depression, L – level, LN_Sigma and LN_Omega are error terms and not used in getting the point estimate of the utility.*

1. **Supplemental Results**

**Supplemental Table 2.** Fit Statistics On Individual Level Observations

| Out of sample fit statistics | Regular sigma | | | | | | |  | Heteroscedastic sigma | | | | | | |
| --- | --- | --- | --- | --- | --- | --- | --- | --- | --- | --- | --- | --- | --- | --- | --- |
|  | Fixed intercept | | |  | Random intercept | | |  | Fixed intercept | | |  | Random intercept | | |
|  | TTO |  | Hybrid |  | TTO |  | Hybrid |  | TTO |  | Hybrid |  | TTO |  | Hybrid |
| RMSE | 0.3177 |  | 0.3178 |  | 0.3177 |  | 0.3176 |  | 0.3222 |  | 0.3242 |  | 0.3211 |  | 0.3228 |
| MAE | 0.2244 |  | 0.2270 |  | 0.2244 |  | 0.2262 |  | 0.2300 |  | 0.2321 |  | 0.2293 |  | 0.2312 |
| CCC | 0.7616 |  | 0.7610 |  | 0.7617 |  | 0.7615 |  | 0.7311 |  | 0.7243 |  | 0.7357 |  | 0.7291 |
| ICC | 0.7616 |  | 0.7610 |  | 0.7617 |  | 0.7615 |  | 0.7311 |  | 0.7243 |  | 0.7357 |  | 0.7290 |
| R | 0.7836 |  | 0.7834 |  | 0.7836 |  | 0.7837 |  | 0.7822 |  | 0.7806 |  | 0.7826 |  | 0.7816 |

Notes: TTO – time trade off only model, Hybrid – time trade off and discrete choice model, RMSE – root mean square error, MAE – mean absolute error, CCC - Lin’s Concordance Correlation Coefficient, ICC - Fisher’s Intraclass Correlation Coefficient, R - Pearson’s R

**Supplemental Table 3.** Out of sample log-likelihood on Individual Level Observations

|  | Regular sigma | | | | | | |  | Heteroscedastic sigma | | | | | | | | | |
| --- | --- | --- | --- | --- | --- | --- | --- | --- | --- | --- | --- | --- | --- | --- | --- | --- | --- | --- |
|  | Fixed intercept | | |  | Random intercept | | |  | Fixed intercept | | |  | Random intercept | | | | | |
|  | TTO |  | Hybrid |  | TTO |  | Hybrid |  | TTO |  | Hybrid |  | TTO | |  | Hybrid | | |
| Block_1 | -241,812 |  | -240,970 |  | -165,334 |  | -166,806 |  | -17,825 |  | -15,292 | |  | 25,834 | | |  | 27,243 |
| Block_2 | -273,063 |  | -277,729 |  | -148,083 |  | -153,128 |  | -120,446 |  | -120,698 | |  | -83,240 | | |  | -83,111 |
| Block_3 | -250,916 |  | -255,449 |  | -166,988 |  | -172,502 |  | -149,986 |  | -142,748 | |  | -114,552 | | |  | -112,035 |
| Block_4 | -293,672 |  | -292,727 |  | -223,171 |  | -221,570 |  | -121,202 |  | -116,252 | |  | -106,490 | | |  | -101,426 |
| Block_5 | -248,308 |  | -235,824 |  | -146,417 |  | -130,538 |  | -78,479 |  | -75,480 | |  | -57,565 | | |  | -54,166 |
| Block_6 | -279,791 |  | -284,341 |  | -232,353 |  | -236,534 |  | -190,222 |  | -178,406 | |  | -163,756 | | |  | -158,577 |
| Block_7 | -177,158 |  | -177,513 |  | -127,475 |  | -127,189 |  | 8,095 |  | 3,389 | |  | 36,455 | | |  | 32,058 |
| Block_8 | -109,521 |  | -116,463 |  | -47,394 |  | -53,148 |  | 15,569 |  | 6,643 | |  | 54,999 | | |  | 46,493 |
| Block_9 | -310,802 |  | -308,331 |  | -176,947 |  | -174,205 |  | -219,365 |  | -216,884 | |  | -178,515 | | |  | -179,209 |
| Block_10 | -230,031 |  | -228,701 |  | -147,352 |  | -144,625 |  | -17,437 |  | -19,950 | |  | 31,170 | | |  | 30,627 |
| SUM | -2415,075 |  | -2418,047 |  | -1581,513 |  | -1580,245 |  | -891,298 |  | -875,679 | |  | -555,661 | | |  | -552,104 |

**Supplemental Table 4.** Fit Statistics On State Means

| Out of sample fit statistics | Regular sigma | | | | | | |  | Heteroscedastic sigma | | | | | | |
| --- | --- | --- | --- | --- | --- | --- | --- | --- | --- | --- | --- | --- | --- | --- | --- |
|  | Fixed intercept | | |  | Random intercept | | |  | Fixed intercept | | |  | Random intercept | | |
|  | TTO |  | Hybrid |  | TTO |  | Hybrid |  | TTO |  | Hybrid |  | TTO |  | Hybrid |
| RMSE | 0.0654 |  | 0.0655 |  | 0.0653 |  | 0.0645 |  | 0.0828 |  | 0.0897 |  | 0.0788 |  | 0.0849 |
| MAE | 0.0507 |  | 0.0529 |  | 0.0506 |  | 0.0516 |  | 0.0601 |  | 0.0627 |  | 0.0578 |  | 0.0603 |
| CCC | 0.9860 |  | 0.9860 |  | 0.9861 |  | 0.9864 |  | 0.9748 |  | 0.9699 |  | 0.9775 |  | 0.9733 |
| ICC | 0.9860 |  | 0.9860 |  | 0.9861 |  | 0.9864 |  | 0.9748 |  | 0.9699 |  | 0.9775 |  | 0.9733 |
| R | 0.9861 |  | 0.9861 |  | 0.9862 |  | 0.9865 |  | 0.9846 |  | 0.9827 |  | 0.9851 |  | 0.9839 |

Notes: TTO – time trade off only model, Hybrid – time trade off and discrete choice model, RMSE – root mean square error, MAE – mean absolute error, CCC - Lin’s Concordance Correlation Coefficient, ICC - Fisher’s Intraclass Correlation Coefficient, R - Pearson’s R

**Supplemental Table 5.** Out of sample log-likelihood on State means

|  | Regular sigma | | | | | | |  | Heteroscedastic sigma | | | | | | |
| --- | --- | --- | --- | --- | --- | --- | --- | --- | --- | --- | --- | --- | --- | --- | --- |
|  | Fixed intercept | | |  | Random intercept | | |  | Fixed intercept | | |  | Random intercept | | |
|  | TTO |  | Hybrid |  | TTO |  | Hybrid |  | TTO |  | Hybrid |  | TTO |  | Hybrid |
| Block_1 | 1,992 |  | 1,983 |  | 3,002 |  | 2,967 |  | 3,428 |  | 3,479 |  | 3,844 |  | 3,861 |
| Block_2 | 2,231 |  | 2,152 |  | 3,104 |  | 3,030 |  | 3,599 |  | 3,586 |  | 3,995 |  | 3,973 |
| Block_3 | 2,090 |  | 2,021 |  | 3,002 |  | 2,922 |  | 3,733 |  | 3,733 |  | 4,069 |  | 4,030 |
| Block_4 | 2,179 |  | 2,166 |  | 3,174 |  | 3,165 |  | 3,544 |  | 3,517 |  | 3,944 |  | 3,921 |
| Block_5 | 1,950 |  | 2,079 |  | 2,733 |  | 2,908 |  | 3,666 |  | 3,751 |  | 4,046 |  | 4,119 |
| Block_6 | 2,222 |  | 2,149 |  | 3,236 |  | 3,166 |  | 4,172 |  | 4,188 |  | 4,451 |  | 4,477 |
| Block_7 | 1,993 |  | 1,970 |  | 2,982 |  | 2,970 |  | 3,650 |  | 3,589 |  | 4,119 |  | 4,031 |
| Block_8 | 2,035 |  | 1,937 |  | 3,026 |  | 2,939 |  | 3,922 |  | 3,771 |  | 4,350 |  | 4,178 |
| Block_9 | 2,177 |  | 2,183 |  | 2,987 |  | 3,007 |  | 3,406 |  | 3,404 |  | 3,851 |  | 3,809 |
| Block_10 | 2,196 |  | 2,188 |  | 3,133 |  | 3,138 |  | 4,407 |  | 4,273 |  | 4,849 |  | 4,706 |
| SUM | 21,064 |  | 20,829 |  | 30,380 |  | 30,212 |  | 37,526 |  | 37,290 |  | 41,518 |  | 41,106 |

**Supplemental Table 6.** Distribution of Domain(s) Considered when Comparing Health States with Different Lengths of Life during the Discrete Choice Experiment, EQ-5D-5L VT Survey, Philippines, 2017

| **Domain** | **Count** | **% (n=1000)** |
| --- | --- | --- |
| Mobility | 748 | 74.8 |
| Self-care | 763 | 76.3 |
| Usual Activities | 668 | 66.8 |
| Pain/Discomfort | 549 | 54.9 |
| Anxiety/Depression | 451 | 45.1 |
